# Meta-Decision in Healthcare

**DOI:** 10.3389/fpubh.2021.694689

**Published:** 2021-06-15

**Authors:** Latifa Mohammad Baynouna Al Ketbi

**Affiliations:** Abu Dhabi Healthcare Services, Al Ain, United Arab Emirates

**Keywords:** meta-decision, decision-making, triple aim, quality care, evidence-based medicine, value of health

## Abstract

Meta-decision as a junction between evidence and its rightful implementation is suggested in this review as a structured framework applied in healthcare, valuable to clinicians and healthcare decision-makers. The process of meta-decision requires optimum measurements to provide data necessary for identifying and developing decision alternatives and explicitly reflect on its value and choose the optimum decision. The location of value in the meta-decision framework is core component. Of equal importance are prerequisites for decision-makers' abilities to make meta-decisions and focus on optimum team environments. As well as improving their decision-making process through reflection and learning.

## Highlights

- What do we already know about this topic?

The decision-making process and Meta - decision was much more studied in other disciplines than healthcare. The incorporation of value in decision making from both the patient and healthcare providers prospective in decision making is increasingly identified as an essential area of research.

- How does your research contribute to the field?

The meta-decision concept contributes by highlighting bias affecting the decision-making process and the importance of the decision maker capabilities. It facilitate verbalization, auditing and teaching and widen learner prospective through stimulating directed search in multiple domains rather than one-line inquiry of effectiveness “is it right for the patient/individual, community/population and for me as decision maker?”.

- What are your research's implications toward theory, practice, or policy?

The Meta - decision is a structured approach to decision-making in healthcare suggested to reduce inappropriately basing care on deficient perspectives or evidence. Meta-decisions' competency prevents an incorrect decision's consequences, uncovers gaps in meta-decision-making, and provides a method for assessment and training for that purpose.

## Background

Decision-making is crucial for safe and effective healthcare practice. It is required to promote the best clinical and public outcomes—cures and optimum healing that are efficient, cost effective, and patient-centered. Inappropriate or uncertain decisions result in low-quality care and delay best-evidence implementation. However, decision-making uncertainty is not always caused by lack of evidence; it is increasingly caused by challenges posed by multiple options with similar comparability and efficiency as tremendous advancements in medicine have increased the number of options and their complexity. The value of these options varies in different contexts, among different patients or populations, and according to decision-makers' abilities and values.

In the last few decades, evidence-based medicine (EBM) has facilitated decisions based on the integrating of research evidence, personal experience, and patients' preferences. Nevertheless, EBM has been challenged for being misappropriated by vested interests and being less patient-centered, having a large information load, less clarity on options' clinical significance, and guidelines that are difficult to tailor to complex multi-morbidities (1). For EBM to overcome these challenges, more efforts must be exerted to form a junction between knowledge and practice. Since an EBM question has a singular answer to the question about an option's efficiency/effectiveness, applying it in practice necessitates determining the value of the option, which may require many questions being answered in multiple domains to make a decision. Therefore, decision-making is the junction and must be studied and improved for better outcomes.

Unlike in other disciplines, the process of choosing from among multiple decision alternatives has not been well-studied in healthcare literature. Kelly et al. (2) highlighted the need to make values explicit, explore them systematically, and integrate them into decision-making, since values are integral to the practice of EBM. According to them, the science of EBM focuses primarily on methods for reducing bias in the evidence, while the role of values in different aspects of the process has been almost completely ignored. Thus, this review proposes a meta-decision framework as an attempt to contribute to this field. The main aim of this framework is planning and determining how to make decisions through a hierarchical structure formation comprising three distinct steps suggested by Simon. (3) Simon (4) stated that “when the problem is simple or when the situation is static, the approaches available to rational decision-makers are acceptable, but the same cannot be said when the situations are dynamic, complex, and involving uncertainties.”

In this adapted framework of meta-decision in healthcare, it is suggested that “value” is based on the well-described and identified criteria of the important outcomes in healthcare—the triple aim. It was hypothesized as an approach to facilitate the planning of interventions and ensuring cost effectiveness and patient-centered care. However, achievement of one aim should not come at the expense of the remaining two aims. Berwick et al. (5) argued for addressing these dimensions simultaneously to deliver the desired outcomes. Nevertheless, despite many reports of successful Triple Aim implementations, commonly used measures often differ and fail to capture all its domains (6–8).

With data increasingly being generated through the wide use of electronic health records in healthcare at the patient care and healthcare management levels, and for financing and quality improvement with instant processing at point-of-care. It is anticipated that the study of meta-decisions in healthcare will be of assistance in this field through the identification of gaps and suggestion of solutions (9). Additionally, this approach will allow for generating knowledge on managing artificial intelligence data. Here, the initiation starts with a need for meta-decision frameworks that when applied can result in efficient processing and better outcomes, avoiding blind data mining and unstructured data management. This approach can be automated in steps following the meta-decision processes with interruptions and cycles as needed.

Finally, it is necessary to conduct studies that help in understanding the dynamic aspects of decision-makers' behavior during the decision-making process (10). Many meta-decision case studies in healthcare reporting their success and failure can help promote learning and knowledge transfer for practice and facilitate use of the best evidence.

This review discusses the conceptualization relevance and application of meta-decisions in healthcare, with emphasis on the prerequisites of the decision-maker, growing demand for detailed measurements, and the context of meta-decisions' implementation, along with examples.

## Meta-Decision: History and Applicability In Healthcare

Several studies have examined the concept of meta-decision (9–11). Wang (9) defined it as “the decision on how to make the practical decisions required throughout the whole decision process.” Mintzberg (11) called it program control, an overall process of planning and switching in decision-making, and acknowledged the difficulty of the process stating that “decision control activities are difficult to study because they tend to be implicit and informal, taking place in the mind of the decision-maker and to leave little trace of themselves.”

The meta-decision concept was used more in non-healthcare models, but despite its importance, the literature on this subject is limited and dispersed over time (10, 12, 13). Thus, it is worthwhile to conceptualize and propose its implementation in healthcare, and acknowledge that it is based on successful experiences in non-healthcare literature (14, 15). They identified distinct steps for decision-making, which are very similar but differ in number of steps and terminology (11, 14, 16).

The meta-decision steps include rationality as an important concept. It strongly relates to Simon's bounded rationality theory, which indicates that rationality is limited when individuals make decisions based on the tractability of the decision problem, cognitive limitations of the mind, and available time. Decision-makers, in this view, act as satisfiers, seeking a satisfactory rather than an optimal solution (3, 16–18).

Several concepts and tools are described in healthcare to facilitate evidence-based decision making, especially at the population or policy level. Many of them overlap in some parts or are very similar. It can be grouped into two categories: first, research data analysis and appraisal or analysis and the second is development of value assessment criteria. the first include meta-analyses, network meta-analyses, and comparative effectiveness research which search, aggregate, and appraise studies to produce and/or compare outcomes (19). Mathematical modeling is increasingly being used in such studies (20). The depth usually addresses one aspect (21) and generates answers to a single domain, such as effectiveness or cost, and is less often inclusive of patients'/populations' values or environmental and setting issues. Additionally, conclusions may not be easily transferable to practical policies or be appropriate for clinical and economic decision making. This is due to limited generalizability or relevance to real-world clinical settings and other limitations. Furthermore, the analyses include only measurable factors (21).

Examples of the second category “value assessment during decision making” include multi-criteria decision analysis (MCDA) (22, 23) and Decision Makers' Program Planning Tool (DMPPT) (20). The evidence-informed deliberative processes framework is another framework which is described as a hybrid value assessment framework of both accountability for reasonableness, A4R, and MCDA (24). These frameworks is a core aspect of various bodies, including the International Society of Pharmacoeconomics and Outcomes Research (ISPOR), the Academy of Managed Care Pharmacy (AMCP), and the National Pharmaceutical Council (NPC) which advocate efforts to provide tools for evidence-based decisions in health care.

In this study, the meta-decision will be supported with examples from patient care and policy levels. It is followed by expanded discussions on value and bias, fundamental concepts within this framework.

## The Meta-Decision Framework

To the best of our knowledge, no similar discussion has been done in the healthcare literature. Although it is beyond the scope of this study to describe how the concepts of decision-making were developed and how different frameworks (11, 14, 16–18, 25) relate to each other, the focus is a simplified adaptation and applicability of this framework to healthcare that stimulates reflection and learning.

**Figure 1:**
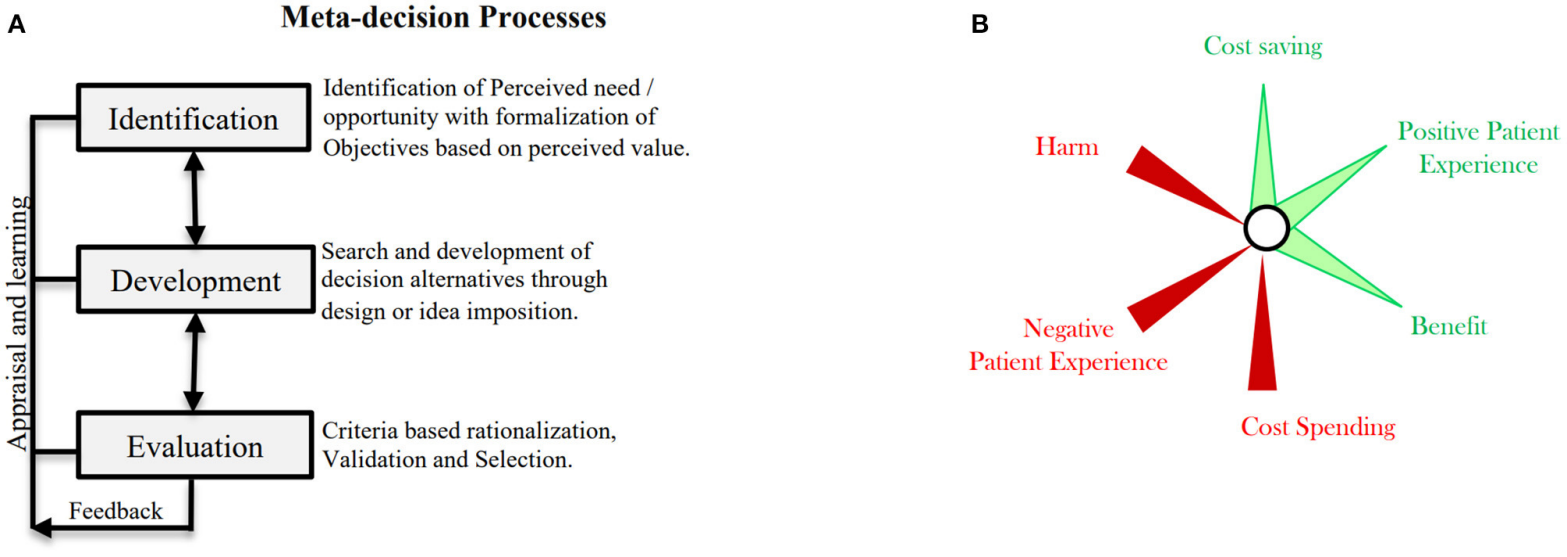
The meta-decision framework. **(A)** The framework steps. **(B)** Reflection on values of importance which are four dimensions, benefit, harm, cost, and personal Experience.

**Figure 2:**
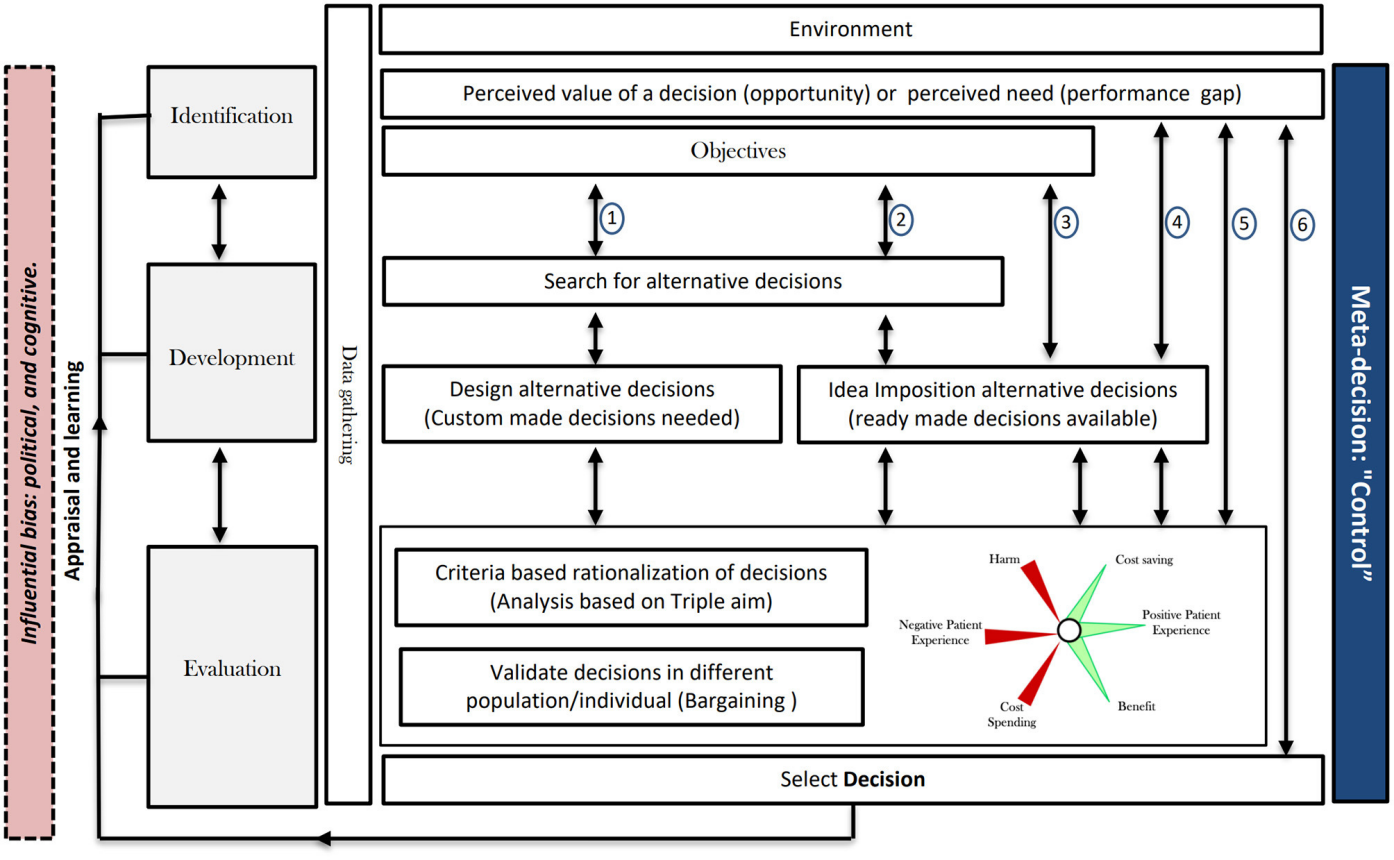
Examples from healthcare to illustrate the meta-decision framework application. These examples suggest variants of the meta-decision processes. In possibility 1, all of the mentioned steps progress in sequence, including a design development option. Possibility 2 is similar, but instead of designing custom-made ideas, a ready-made idea is used. In possibility 3, no search takes place and ready-made ideas are sought directly. In possibility 4, the decision maker skips the formulation of aims and the search; based on the identification, he/she moves directly to the evaluation phase. In possibility 5, the decision maker moves from identification to selection where the development phase is omitted. In possibility 6, all steps from the identification to the decision are skipped. The evaluation step could be different in all of these case studies with regard to the population studied and their economic status, educational level, and age. Research using data collected locally is crucial for making informed decisions for all special populations in all settings as well as for validation of decisions.

As shown in Figure 1, the concept of meta-decision generally follows three distinct steps: identification, development, and evaluation. Through the introduction of these steps, two examples will be used to apply the meta-decision concept in detail—one at the patient care level and the other at policy level. Additional examples are listed to further clarify the concept in Figure 2.

### Identification

In the identification step, there is either a need for a decision or a perceived value of a decision (opportunity). A need has a performance gap, measuring how far one is from deciding, which helps in planning the decision-making. With an opportunity, there is already a decision to consider. Mintzberg et al. (11) states, “Perhaps opportunities do not require much investigation there is nothing to correct, only something to improve.” The application of this step necessitates an understanding of the environment, mainly, the timeframe, context, and population.

*Example (1) - patient care level:*

*A 30-year-old man attends his first preventive care visit. Other than positive family history for ischemic heart disease, slightly raised lipid profile, and smoking, he has no other illness. The attending physician is contemplating lipid-lowering medications*.

*The use of statin to target lipid levels and decrease cardiovascular events is a decision made by the treating physician. Since the patient is currently low risk, the risk of a cardiovascular event occurring in the next 10 years is*<*2.5%. Nevertheless, if the patient were high risk, 65-years-old, and with high blood pressure, the risk of a cardiovascular event in the next 10 years would be more than 25%. This is an opportunity decision*.

*Example (2) - policy level:*

*Introducing genetic testing in premarital screening. It is necessary to decide whether there is value in universal screening for the community and individual couples*.

Understanding the needs and opportunities would help initiate data collection early and formulate the objectives. Rationality necessitates the incorporation of values early on. Moreover, as discussed, meta-decisions are ideally conceptualized in healthcare based on the triple aim. There is great value in placing the triple aim's components in relation to each other from the start of the process of decision-making, meta-planning, and purposeful comprehensive planning if possible, to achieve outcomes that matter. This may help in early visualization of any identified achievements or gaps produced.

*Example (1). Areas of identified objectives for prescribing statin relates to longevity, financial burden, decreasing cardiovascular events, and patients' perceived social, physical, psychological and environmental values. Nevertheless, all could differ due to varying patient risk profiles*.

*Example (2). The policy-level identified objectives for implementing genetic testing at premarital visits include lowering disease burden, suffering, and death due to fewer congenital diseases, and social and cultural acceptance of testing and their consequences. Possible generated data use could be a secondary objective to inform patient care and policy, such as in precision medicine development. There are also important objectives protecting patients' privacy, avoiding exposure of genetic information, financial requirements planning, and community acceptance*.

### Development

In the development step, alternatives are sought. Searching for an easy, readymade decision idea imposition process or a search and discovery process is required if no ready solution is available or not seen as suitable by the decision-maker, which necessitates the design of custom-made decisions or innovation.

*Example (1). Statin is used for primary prevention* (26), *its use has been studied extensively, and well-developed guidelines exist to support decision-making. Nevertheless, there are reports of deficiencies in reporting harm* (27). *Therefore, the development of decision options requires research and credible references. Differences in patients' risk profiles and contexts necessitates the development of alternatives before making decisions. Possible decision alternatives could include starting with lifestyle changes and low- or moderate-intensity statins or the same first step and adding statin if lipid therapeutic targets are not met. Another alternative is to start with high-intensity statin or a combination of more than one lipid-lowering medication. Based on data from the identification step, decision options are altered. If the patient has additional risk such as chronic kidney disease, the search will be expanded to adapt alternatives for this additional risk*.

*Example (2). Decision-makers at the policy level for premarital genetic disease screening may/ may not have sufficient guidelines to detail all target conditions. If available evidence is not organized in recommendations for specific conditions, but rather for the most common ones, such as hemoglobinopathies, solutions will have to be developed. Options are conducting selective screening for certain population subgroups such as certain ethnicities, patients with family or suggestive history, or all populations, to conduct screening for selective diseases found to have stronger evidence, or not screening at all*.

### Evaluation

In the evaluation step, alternative decisions are evaluated, the optimum decision is selected, and resources are allocated for implementation. It is a critical phase involving analysis, bargaining, and judgement. Mintzberg et al. (11) mentioned that in the evaluation or selection phase, analysis can distinguish between facts and value. Bargaining, on the other hand, is dependent on the context and decision-maker/end user perception of value and in judgment, the end-user values are determinant to balance options against each other and should be considered in rationalizing optimum shared decision-making.

Figure 1B shows the suggested criteria to rationalize and judge value in the evaluation step based on the triple aim domains where achieving optimal target outcomes in each domain is dependent on considering the context, population difference, and short- and long-term outcomes, all of which would collectively converge at the bullseye. The bullseye represents “the ideal target/outcome,” that is, the outcome that results in maximum benefit and optimal safety with minimal costs and maximal care experiences for both individual patients and the population.

*Example (1). The physician might consider that although statin can be used for high-risk and low-risk patients, for the former, the benefit far outweighs the potential harm. Harmful side effects, could include short-term muscular pain and a long-term risk of diabetes. However, harm occurrence depends on harm rarity in different age groups. Moreover, ethnicity is a determinant of diabetes, therefore, its occurrence probability will vary accordingly. The target population's vulnerability regarding comorbidities—liver or kidney diseases—is to be considered, and patients' tolerance of the drug is also unpredictable. Furthermore, in countries with fewer resources, the cost per capita of healthcare may limit statin use, and healthcare-related decisions may not that of resource-rich countries. Thus, identified gaps in equity necessitate suitable plans: the decision for higher-risk patients might be high-intensity statin while for lower-risk patients, counseling him/her about positive family history and his/her preferences regarding his/her ability to change and health priorities*.

In this situation, in which a patient reflects on his/her values of what he/she sees as a gain or loss and the consideration of acceptability of lifestyle modifications vs. commitment to lifelong statin treatment with an emphasis on the long-term benefits, harms and costs are determinants in the bargaining process, adding to the physician's rationality of determining value. Preferably, the patient will be informed about their options and will contribute to the decision by weighing options with the physician. In the end, the shared best judgement between the physician and patient is selected. However, when identifying any need to change or any new opportunity to improve a patient's life, this new identified need or opportunity is followed by the development of and search for new possible options, after which re-evaluation occurs and multiple meta-decision cycles may be needed to reach the best decision.

*Example (2). Validating decisions for a national genetic screening program will require information on the budget and resources needed along with local data on genetic diseases' epidemiology, socio-economic status, and health literacy—essential to inform decision consequences. Careful analysis of each decision developed previously is to be validated through bargaining and weighing options against each other. For example, while genetic testing has the long-term benefit of avoiding genetic diseases in children, a short-term outcome may be test anxiety due to results of uncertain significance or any immediate decisions the couple may take to avoid children with possible diseases. Another long-term harm is exposing the couple and their children to explicit genetic data with possible employment or treatment disadvantages. The freedom to make choices might be affected due to the influence of others, such as the government or their community, on decision-making*.

The process appraisal and feedback on the success or failure of meta-decisions' processes contribute to improving future meta-decisions. Research using local data is crucial for making informed validated decisions for special populations in all settings. The approach of meta-decisions will also prevent unstructured decision-making and accommodate differences.

It is worth noting that clinical practice guidelines and protocols provide choices, facts, and ideas that can be used in the meta-decision process and its steps. Therefore, meta-decision is the junction to deliver the right evidence to the right patient. This is true for clinical decision support tools as well, which are increasingly available at the point of decision-making. It was found to reduce costs, improve quality, and reduce medical errors in clinical settings (28). Nevertheless, it provides mainly clinical knowledge, but is not relevant to other domains considered important in medical decision-making as a social determinant of health and patient preferences (29). However, the question remains of does it facilitate accessibility to data and ideas on expenses of limiting search and design. This needs to be better studied through its use with the meta-decision approach. Table 1 shows the concept applied on more examples.

**Table 1:** Relevance of meta-decision approach in decision making in healthcare. Practice and policy perspectives.

**Step**			**Individual level**	**Policy level**	**Individual level**	**Policy level**
Evaluation
	Identification		Lipid-lowering medication (statin) used for primary prevention (performance gap)	Introducing law on banning e-cigarettes smoking (opportunity)	New home glucose monitoring program for diabetic patents (opportunity)	Pre-marital genetic disease screening (performance gap)
	Development		Determine multiple possible decisions using either: *Search*			
	*Rationalize*: (Analysis and bargaining)		1. Ready-made. For example, from research studies, Clinical Practice Guidelines, protocols, policy, or other sources *Search and design* (innovate)			
			2. Custom-made. For example, by task force, team discussions, experts' or leaders' opinions, or individually built options			
		Benefit	Decrease in cardiovascular (CVD) outcomes	Decrease in smoking, increase in community awareness	Decrease in hypoglycemia, better control, better CVD outcomes	Decrease in hereditary diseases
		Harm	Evidence of reported and unreported harm	Economic impact.	Worse quality of life with frequent testing	Worse quality of life due to anxiety over tests results of uncertain significance, and risk of immediate decisions due to fear of consequences of positive results
		Cost	High	Cost saving (decreases in smoking prevalence and related diseases)	High	High
		Patient Experience:	Side effects acceptance, preventive proactive attitude	Community acceptance due to perception of coercion and affected patient autonomy	Variable either favorable or not.	Community acceptance due to perception of coercion and affected patient autonomy
	*Validation*:		To consider age, conditions' severity, ethnicity, socio-economic state,Long- and short-term outcomes	To consider cultural and health long- and short-term outcomes	To consider diabetes control status, health literacy, age, medication type,long- and short-term outcomes.	To consider genetic diseases' epidemiology, socio-economic state, health literacy, long- and short-term outcomes
		**Judgment and decision-making**					

## Sequence of The Meta-Decision Process

All frameworks on which the proposed framework is based suggest that as decisions often require subsequent modification or even complete redesign, the sequence is important but not essential.

Therefore, a switch in decisions can occur through interruptions—sudden events that obstruct the process. Moreover, iterations may occur, wherein faced with a failure, the decision-maker cycles back to an earlier phase to understand needs by gathering more data or develop alternative decisions

Consequently, the main aim of meta-decision-making is the formation of a hierarchical structure that improves the quality of decision-making in practice but does not create “recipes” requiring simple repetition (9).

Finally, the timeline and pressure affect the processes. Decisions are made daily, some immediately while others, over months or years. Over time, there is movement across the meta-decision process. Mintzberg et al. (11) applied the concept to multiple examples, such as the introduction of new medication in a hospital. Over 2 years, the decision-making process went through the abovementioned three steps but encountered multiple interruptions and multiple development cycles and evaluation/selection steps. Political bias was also reported as affecting decision-making. Figure 2 suggests more examples with different possible meta-decision processes. The minimum process comprises identification and evaluation. With increasing complexity, all meta-decision steps, with many interruptions and cycles, can occur.

### Value

Constructing generic health value measures may not be possible (30), but assessing the value of health is more appropriate and having a framework like the triple aim provides a simple and comprehensive approach. With the complexity of measuring consequences of disease and the total value of health over various contexts, starting with pre-set measures to support decision-making is impossible (30). This is complicated by substantial variability in reported healthcare data due to factors such as clinical inertia, patient expectations, and financial capacity, which differ greatly among different cultures and countries. It is therefore suggested to introduce value early in the decision-making process and work upwards from available data to develop decision options. Consequently, determining the best value relies on data gathering and adapting to local factors.

If data on effectiveness, harm, cost, and patient experience is deficient, then it may be challenging to clearly state the optimal decision. Hendrikx et al. (8) conducted an international comparative analysis to assess which triple aim measures are being used to evaluate population management (PM) initiatives. Of the 865 measures used by 20 PM initiatives, only 11 PM initiatives included all qualities of care domains. However, each triple aim domain has challenges for optimum judgment to be made at any of the meta-decision steps. First, improving the health of populations through healthcare has been measured by a limited number of studies. A well-known measure is the Center for Disease Control's Summary Measures of Population Health (31), which combine information on mortality and non-fatal health outcomes to represent population health in a single number. Another example is the quality-adjusted life year (QALY) and the disability-adjusted life years, which were developed as measurement units to quantify the burden of disease and injury on human populations (32). Challenges with such measures are that they are as accurate as the data sources and context from which they were derived. The variation of healthcare systems, payment structures, and patients' determinants of health is what makes data deficient (33). Therefore, it may not be suitable for generalizability, and may not have the statistical accuracy required to confidently estimate the desired outcomes. Additionally, the available metrics are never comprehensive enough to assist in all decision areas in any context's unique details. Further, a major barrier for QALY is that it is assigns a weight between 0 (for death) and 1 (100% health) to each health state and then multiplies that value by how long the state lasts (34). This method provides a crude idea for policy decision-making, but it is difficult to apply at the patient level.

Regarding cost, it is changing, unsustainable, and not unified for each encounter, patient, or setting. For example, the financial consequences after a myocardial infarction in an adult male varies with different influences on a patient's life, family, work, and medical resources utilized. Less attention is paid to eliminating wasteful spending such as missed prevention, unnecessary service, inefficiently delivered care, high-priced services, excess admirative costs, and fraud—applicable to individual and population levels.

An even more challenging area in the value of health is patient/population values assessment. There are conflicting reports on the relationship between positive patient experience and patient outcomes (35), which is beyond the scope of this review to investigate. Nevertheless, valuing health is only complete with patient perspectives, and their judgment of value is best when all relevant information is available, free of rational flaws like self-interest. Assessment of such judgment was suggested by using personal experience over personal preference due to preferences affecting judgment, or “being guilty of wanting something that could be detrimental” (36). A more practical and simpler approach was then suggested by rationing health care “in terms of how severely they limit the range of valuable lives individuals can live in just two dimensions: activity limitations and health-related feelings” (37).

The subjective nature of health and well-being ratings by patients may be biased. For example, the immediate emotional reactions could be misleading compared to the overall and long-term outcome. Therefore, deliberative focus groups rather than individual surveys should be used for such judgments (37).

Reflecting on population value is different from individual-level value. The population-level value is usually permanent, and the welfare of a country, context, and population diversity greatly contributes to it. Contribution to the judgment is the extent that the individual is involved in decision-making vs. the government. For example, some countries see the introduction of colorectal screening programs by the government as a necessity, while others do not even if the average population preference is supportive.

Decisions regarding population needs are dependent on the principles that governments adopt when they prioritize alternative health programs. Should governments adopt minimal principles and leave decisions to individual self-motivation, or should it implement social goals and expectations for the population? Should the government use coercion to ensure participation in health programs, or should it use coercion and information? Answering any of these questions needs information (38). Hausman described how we may reflect on the policy level of these principles: “the welfarist approach where one thinks of the government as everybody's mother with advancing individual welfare might be requiring intrusions into individual life. Or liberals who regard the government as a protector, insurer, and arbitrator but not as an active partner in individual pursuits. The former may promote passivity in choosing personal priorities in health” (37). These preferences for alternative ethical principles are called meta-preferences (38). The best approach is probably carefully demarcating the line between not harming and not being harmed in the bargaining in the evaluation step. It is also important to consider that public value cannot be sensitive to all relevant details, nor can they be accurately measured. Therefore, implementing meta-decision while reflecting on patients' choices, will build data to inform better future decisions with adaptation to each country's norms.

Finally, an essential component of meta-decisions based on the triple aim is the consideration of possible harm, which is essential when assessing population health. Harm cannot be reported without being clearly measured and weighed against effectiveness to assess net improvement. Potential benefits are not meaningful without the knowledge and quantification of harmful impacts. Interventions provided by healthcare services are administered with the best of intentions; nevertheless, most inevitably cause harm, ranging from minute to significant. Failure in transparently informing end users of health interventions' potential impacts is inconsistent with the ideals in the triple aim. However, safety assessment is not easily separated from that of effectiveness.

A challenge of focusing on measures but not the overall value is evidenced from examples in healthcare where achieving value is worryingly not always the target but achieving individual measures is. For example, it has been reported that when improvements as a result of organizations' strategies for quality improvement described by the domains of the triple aim affected revenue in for-profit organizations with a decrease in patient visits or orders, sustainability of these strategies were challenged (39). Similarly, a payment system initiative used surrogate measures, such as hospital re-admission for heart failure instead of the target-improved outcome, resulting in negative patient outcomes due to decrease in needed readmissions, raising ethical concerns of implementing what is perceived as best value (39)^1^,^2^. Thus, meeting the target outcome was through developing the wrong choice.

## Bias In Meta-Decisions

Bias affect the conceptualization of research questions, conduction of research, aggregating of evidence, publication, and the point of decision-making. A common bias affecting meta-decision's process is cognitive bias. It is the systematic deviation of people's thinking from rational thought. It affects how questions are asked, aims are determined, alternative decisions are developed, and whether there is rationalization in decision-making. Therefore, cognitive bias is negatively related to the quality of meta-decisions. Moreover, a type of cognitive bias is the outcome bias, wherein a decision is judged as a failure or responsible for one when it leads to undesirable consequences, even if the decision was considered as acceptable prior (40). This is different from hindsight bias, where a decision outcome seems inviable retrospectively (41).

Another relevant cognitive bias is confirmation bias, which is the tendency to search for, interpret, favor, and recall information in a way that confirms one's prior personal beliefs/ hypotheses. Here, the process starts with a decision and not with a performance gap or need. Moreover, in searching for alternatives, only those matching the decision-maker's agenda are sought, and the same applies when rationalizing choices based on the criteria if this stage is reached.

Political bias has an important influence on meta-decision processes. Although it is a very common influential factor, it may negatively affect decisions because it restricts and distorts information flow and draws decision-makers attention from patients, population, or institution goals to their own preferences and self-interests (42). It is noteworthy that a combination of these biases can occur.

## Evidence of Effectiveness

The effect of the decision-making process on outcomes has been better studied in non-healthcare disciplines. Some of these reports show that a rational, analytic mindset overrides the effects of one's intuitions in ethical judgements (40). Moreover, the best decision-making outcomes are characterized by decisions that start with identifying the performance gap as well as the aim/objectives (43). Nutt (44) concluded that the best results are obtained when the search efforts are guided by documented needs based on the quantitative performance gap. This highlights the importance of the identification phase.

A review recommended that decision-makers should adhere to more rational and formalized decision-making processes, resulting in better decision outcomes, noting that different process types' adoption leads to different results (15). In the development phase, the search and design processes are more likely to produce successful outcomes than idea imposition or readymade options (14, 43). While searching, there is a wider exploration for alternatives and longer-term adoption of decisions reached (15). Interestingly, the process of discovery is more successful than the idea imposition process, no matter the urgency, importance, resource level, initial support, decision-maker level, sector, or type of decision. This suggests that crisis meta-decision or meta-decision in acute settings need to follow the same framework.

The effectiveness of incorporating value in decision-making is well-assessed in healthcare literature when relating outcome to value. Rationalization based on value is a target that healthcare literature is aiming at and driving financing toward (45).

## Teaching and Competency

A successful meta-decision is the basis for a transparent decision-making process and creates the decision-making rules—essential for effective discussions and improvement. Additionally, outcomes are easily tracked to decision processes and why they were made. Decision-makers should be aware of the logic and reasoning, rather than intuition and personal bias, during decision-making, as well as how the decision would affect their work outcome. Most importantly, a structured process would ensure learning and make potential future updates and improvements easier. The evidence of effectiveness is gathered to guide practice and obtain best results and appraise failed decisions to reduce harm and improve quality (41). Individuals may be inclined to admit or notice fewer failures than those occurring, and ignoring or concealing failure obscures information essential to learning (43).

Although artificial intelligence can eliminate cognitive and political biases, to date, it cannot replicate good cognitive processes to ensure the best development and rationalization during decision-making. Implications would be a model involving reasoning and rationality based on a study of these processes that would provide decision-making support to users who may lack the necessary skills and on certain occasions. This needs to be included in the curricula of medical graduate schools as competency for practicing healthcare professionals.

## Prerequisites of The Healthcare Provider

Healthcare providers (HCPs) must meet the four prerequisites of being competent, happy, caring, and possessing a strong resolve to achieve. Tracking of these prerequisites may facilitate optimal outcomes regarding individual patient and population health. Additionally, it is clear that there is not always one decision-maker in healthcare. Although it is team-based at the policy level, it is preferably in many situations to share decision-making with patients at the patient level. However, the meta-decision process is shared among the team at for the former and is in the HCP's mind for the latter. Therefore, a HCP who is competent, caring, and having good will is paramount. The first prerequisite, although difficult to measure, is represented in multiple areas of the Accreditation Council for Graduate Medical Education's six competencies: medical knowledge, patient care, professionalism, communication skills, practice-based improvement, and practice-based learning ^3^. Competent HCPs make the right choices to drive practice progress in the right direction. Secondly, care is primarily an attitude that can be demonstrated by a good conscience, humanistic qualities, and altruism. Carelessness commonly describes low performing HCPs who are not motivated to help their patients. One challenge is that attitudes and emotions are not easily measured, so assessments are based on actions. The third prerequisite is to be strong-willed and well-intentioned is key for effective HCPs, independent of the levels of caring or competency. Willingness to change, having a positive attitude, having initiative, and being among initial adopters are the characteristics of high-performing organizations and HCPs. However, the assessment of this prerequisite is likewise difficult and not clearly reported. The fourth prerequisite suggested is part of the “Quadruple Aim” by Bodenheimer (46), aimed at improving the work life of HCPs ^4^. Happy and satisfied HCPs promote wellness and resist burnout, resulting in better and safer care quality for individuals and the population.

## Team Environment

Participation in decision-making is important and was found in studies assessing successful decision outcomes as a positive attribute. More failure was reported in decisions using power for implementation or persuasion in the end process (44). It was found that team who have shared conceptualizations of each other's roles and shared knowledge had better coordination and decision-making. A wealth of empirical evidence is beginning to emerge to support the theoretical arguments concerning the importance of shared mental models to team performance (47). In healthcare, this is obvious in simulation and drills but the group decision-making process need to be evaluated further with regards to steps, rules, dynamics, and coordination.

## Conclusion

Meta-decision is a structured approach for decision-making in healthcare suggested to reduce inappropriately basing care on deficient perspectives or evidence. Meta-decisions' competency prevents an incorrect decision's consequences, uncovers gaps in meta-decision-making, and provides a method for assessment and training for that purpose. Meta-decision is developed based on the successful framework from previous literature and detailed clearly in this study to assist in important healthcare-related tasks. Nevertheless, it needs to be proven from research application in healthcare.

## Author Contributions

The author confirms being the sole contributor of this work and has approved it for publication.

## Conflict of Interest

The author declares that the research was conducted in the absence of any commercial or financial relationships that could be construed as a potential conflict of interest.

## Footnotes

^1^https://catalyst.nejm.org/what-is-value-based-healthcare/

^2^https://www.ncbi.nlm.nih.gov/pmc/articles/PMC4462878/pdf/milq0093-0263.pdf

^3^https://www.acgme.org/Portals/0/MilestonesGuidebook.pdf

^4^http://www.ihi.org/communities/blogs/the-triple-aim-or-the-quadruple-aim-four-points-to-help-set-your-strategy

## Abbreviations

QALY: Quality-adjusted life year
EBM: evidence-based medicine
HCPs: Healthcare providers.
